# Spinal cord stimulation trial can control pain caused by chronic limb-threatening ischemia

**DOI:** 10.1016/j.jpra.2024.09.009

**Published:** 2024-09-14

**Authors:** Yuta Terabe

**Affiliations:** Limb Salvage Center, Kasukabe Chuo General Hospital, 344-0063 midori5-9-4, Kasukabe, Saitama, Japan

**Keywords:** Chronic limb-threatening ischemia, Spinal cord stimulation, Ulcer, Pain, Peripheral artery disease

## Abstract

**Background:**

Patients with chronic limb-threatening ischemia experience long-duration pain during ulcer treatment. Hence, painkillers are gradually increased, but adverse events often occur. Spinal cord stimulation trial is one of the methods used to manage such pain.

**Method:**

The study was performed at the Limb Salvage Center in Kasukabe Chuo General Hospital. Total 41 patients with mean age 70.4 ± 9.58 years underwent spinal cord stimulation trial for 2 weeks. Among them, 33 (80 %) were male, and 8 (20 %) were female. Numerical rating scores, wound results, spinal cord stimulation-related adverse events, and total dose of oral painkillers were evaluated.

**Results:**

Postoperatively, itching and bleeding were reported. The numerical rating scores improved from 7/10 before to 2/10 at 2 days after the spinal cord stimulation (*P <* 0.001). The total doses of oral painkillers showed no change before and after spinal cord stimulation (*P >* 0.05).

**Conclusions:**

Spinal cord stimulation is recommended for peripheral artery disease because it can improve numerical rating scores for a short term. Therefore, this trial approach can sufficiently control pain against chronic limb-threatening ischemia, without undergoing implantation.

## Introduction

Chronic limb-threatening ischemia (CLTI) is the terminal stage of peripheral artery disease (PAD).^1^ CLTI treatment lasts for at least a few months. During this period, patients suffer from pain caused by ischemia and ulcers. Thus, the painkillers are gradually increased and strengthened generally. However, these drugs cause some adverse events.^2^ In particular, melena and clouding of consciousness can cause serious problems, that result in treatment decline.

Spinal cord stimulation (SCS) is a simple procedure with two approaches: puncture trial and implantation wherein 1 or 2 leads are implanted in the epidural space to manage pain. It helps relieve neuropathic pain but not nociceptive pain.^3^ Recently, SCS has been reported to cause an effect on the affective dimensions of pain.^4^

Pain control of CLTI treatment is one of the key points for successful treatment. CLTI patients experience ischemic pain and psychogenic pain due to long-term illness. In this case, management with analgesics often produces adverse events that outweigh the analgesic effect. So SCS can provide early and effective analgesic effects.

Puncture trial was applied in this study. We aimed to assess the ability of the SCS to relieve chronic pain through a puncture trial.

## Materials and methods

Conducted at the Limb Salvage Center, Kasukabe Chuo General Hospital. The inclusion criteria were 1) uncontrolled pain despite taking enough painkillers and 2) ulceration up to grade 2 of the Infectious Disease Society of America classification. Uncontrolled pain was defined as the need to increase the dosage of painkillers, including narcotics, and a persistent numerical rating scale (NRS) of 10. Conversely, the exclusion criteria were as follows: 1) cause of pain other than CLTI; 2) lead insertion failure; and 3) patient's unwillingness.

41 patients were treated with SCS, and the mean age was 70.4 ± 9.58 years. The main comorbidities were diabetic mellitus and chronic kidney disease (G5d) (each: 27 [66 %]) (Table 1). All the patients had CLTI, with three having bilateral CLTI. Majority of the patients only had CLTI (27 [66 %]), while others had comorbidities such as blue toe syndrome (7 [17 %]), vasculitis (6 [15 %]), and Buerger's disease (1 [2 %]).

**Table 1:** Patient characteristics.

Cases (n)	41
Sex (n) (male:female)	33:8
Age (year) (mean, SD)	70.4, 9.58
DM (n)	27
CKD G5d (n)	27
CAD (n)	26
CVD (n)	6
Cancer (n)	2
DL (n)	8
HUA (n)	4
HT (n)	20
LSCS (n)	4
Mental illness (n)	3
COPD (n)	1
ET (n)	1
Dementia (n)	2
CRP (mg/dL) (average, SD)	5.63, 17.5
WBC (10 × 3/μL) (average, SD)	11.1, 15.2

CAD: coronary artery disease, CKD: chronic kidney disease, COPD: chronic obstructive pulmonary disease, CRP: C-reactive protein, CVD: cerebrovascular disease, DL: dyslipidemia, DM: diabetes mellitus, ET: essential thrombocythemia, HT: hypertension, HUA: hyperuricemia, LSCS: lumber spinal canal stenosis, WBC: white blood cell.

The participants underwent a puncture trial for SCS for 2 weeks. Two 8-electrode epidural leads were placed at the top of Th8 and Th10 individually. They were evaluated before and 2 weeks after SCS.

The pain was evaluated by NRS, which was checked at times not related to wound procedures (invasive pain). The wound was classified as healing, major amputation, minor amputation, and death. Other points were SCS-related adverse events and total dose of oral painkillers.

Statistical data were analyzed using the statistical software R, version 4.0.3. For univariate analysis, non-normally distributed data were examined using the Wilcoxon signed-rank test. A P value less than 0.05 was considered statistically significant. The findings were reported in accordance with the STrengthening the Reporting of OBservational studies in Epidemiology (STROBE) checklist.

## Results

All 41 patients took an SCS trial for 2 weeks. No adverse events occurred during the procedure. After the procedure, two mild adverse events (itching and minor bleeding) occurred.

**Figure 1:**
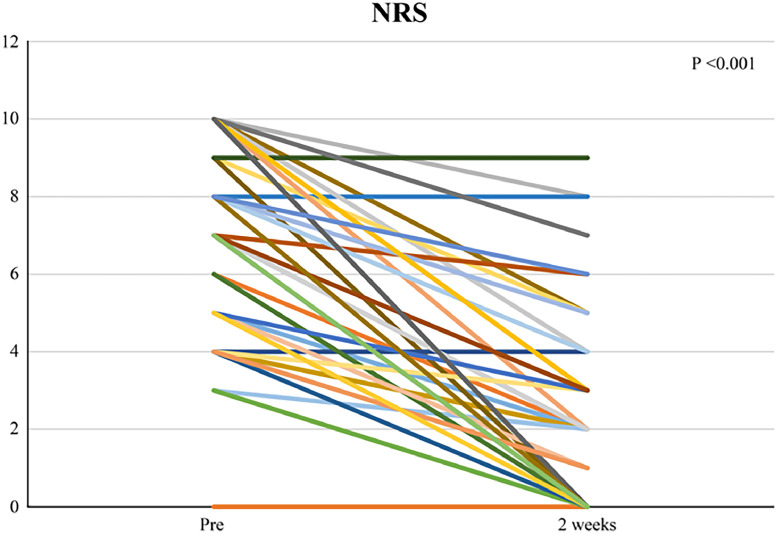
Result of NRS.

The NRS improved from 7/10 before the SCS to 2/10 at 2 days after the SCS (*P <* 0.001) (Figure 1). The total dose of oral painkillers did not change before and after SCS (*P >* 0.05).

The wound healed in 22 (53 %) of the patients but caused major amputation, minor amputation, and death in 9 (22 %), 6 (15 %), and 4 (10 %) patients, respectively.

## Discussion

The treatment period for CLTI is 2 to 3 months with hospitalization in our country. The results of an outpatient CLTI treatment with SCS implantation in long-term observation showed that, compared to the non-SCS treatment group, wound healing rate remained unchanged.^2^ In other words, if the purpose is wound healing, it is thought that there is little advantage in implanting the SCS. Especially, almost all patients with CLTI have foot ulcers, and these patients feel intense pain due to ischemia and ulcer; thus, these patients should be treated immediately.

In the present study, the trial approach is enough to control pain caused by CLTI immediately. In addition, the pain control of the World Health Organization pain ladder will not be ready in time because of severe ischemic pain.^5^ Therefore, when used in combination with SCS trial, adequate pain control was achieved without any adverse events associated with painkillers. This study demonstrates the importance of pain control in the SCS trial to treat hard-to-heal ulcers.

However, NRS did not improve in some cases. It was also found that SCS was not indicated for all painful CLTI cases and improvement in pain does not necessarily lead to wound healing; thus, cases showing SCS effectiveness should be examined in the future.

## Conclusions

SCS is an important method for CLTI pain control, and the trial method is effective for CLTI treatment.

## Compliance with ethical standards

This study was approved by the hospital's research ethics committee (Permission No.2009-1).

## Consent to participate

Participants were explained on the nature of the study before providing written informed consent.

## Presented history

There is no presented history.

## Funding

None.

## Declaration of competing interest

The authors declare that they have no conflict of interest.
